# Analysis of Pharmacokinetics in the Cochlea of the Inner Ear

**DOI:** 10.3389/fphar.2021.633505

**Published:** 2021-05-03

**Authors:** Seishiro Sawamura, Genki Ogata, Kai Asai, Olga Razvina, Takeru Ota, Qi Zhang, Sasya Madhurantakam, Koei Akiyama, Daisuke Ino, Sho Kanzaki, Takuro Saiki, Yoshifumi Matsumoto, Masato Moriyama, Yasuo Saijo, Arata Horii, Yasuaki Einaga, Hiroshi Hibino

**Affiliations:** ^1^ Division of Glocal Pharmacology, Department of Pharmacology, Graduate School of Medicine, Osaka University, Osaka, Japan; ^2^ Department of Chemistry, Keio University, Yokohama, Japan; ^3^ Department of Molecular Physiology, Niigata University School of Medicine, Niigata, Japan; ^4^ G-MedEx Office, Niigata University School of Medicine, Niigata, Japan; ^5^ Department of Otolaryngology, Head and Neck Surgery Niigata University Graduate School of Medical and Dental Sciences, Niigata, Japan; ^6^ Department of Otolaryngology, School of Medicine, Keio University, Tokyo, Japan; ^7^ Department of Medical Oncology, Niigata University Graduate School of Medical and Dental Sciences, Niigata, Japan; ^8^ AMED-CREST, AMED, Osaka, Japan

**Keywords:** sensorineural hearing loss, pharmacokinetics, ototoxic drug, therapeutic reagent, cochlea, real-time measurement

## Abstract

Hearing loss affects >5% of the global population and therefore, has a great social and clinical impact. Sensorineural hearing loss, which can be caused by different factors, such as acoustic trauma, aging, and administration of certain classes of drugs, stems primarily from a dysfunction of the cochlea in the inner ear. Few therapeutic strategies against sensorineural hearing loss are available. To develop effective treatments for this disease, it is crucial to precisely determine the behavior of ototoxic and therapeutic agents in the microenvironment of the cochlea in live animals. Since the 1980s, a number of studies have addressed this issue by different methodologies. However, there is much less information on pharmacokinetics in the cochlea than that in other organs; the delay in ontological pharmacology is likely due to technical difficulties with accessing the cochlea, a tiny organ that is encased with a bony wall and has a fine and complicated internal structure. In this review, we not only summarize the observations and insights obtained in classic and recent studies on pharmacokinetics in the cochlea but also describe relevant analytical techniques, with their strengths, limitations, and prospects.

## Introduction

**FIGURE 1:**
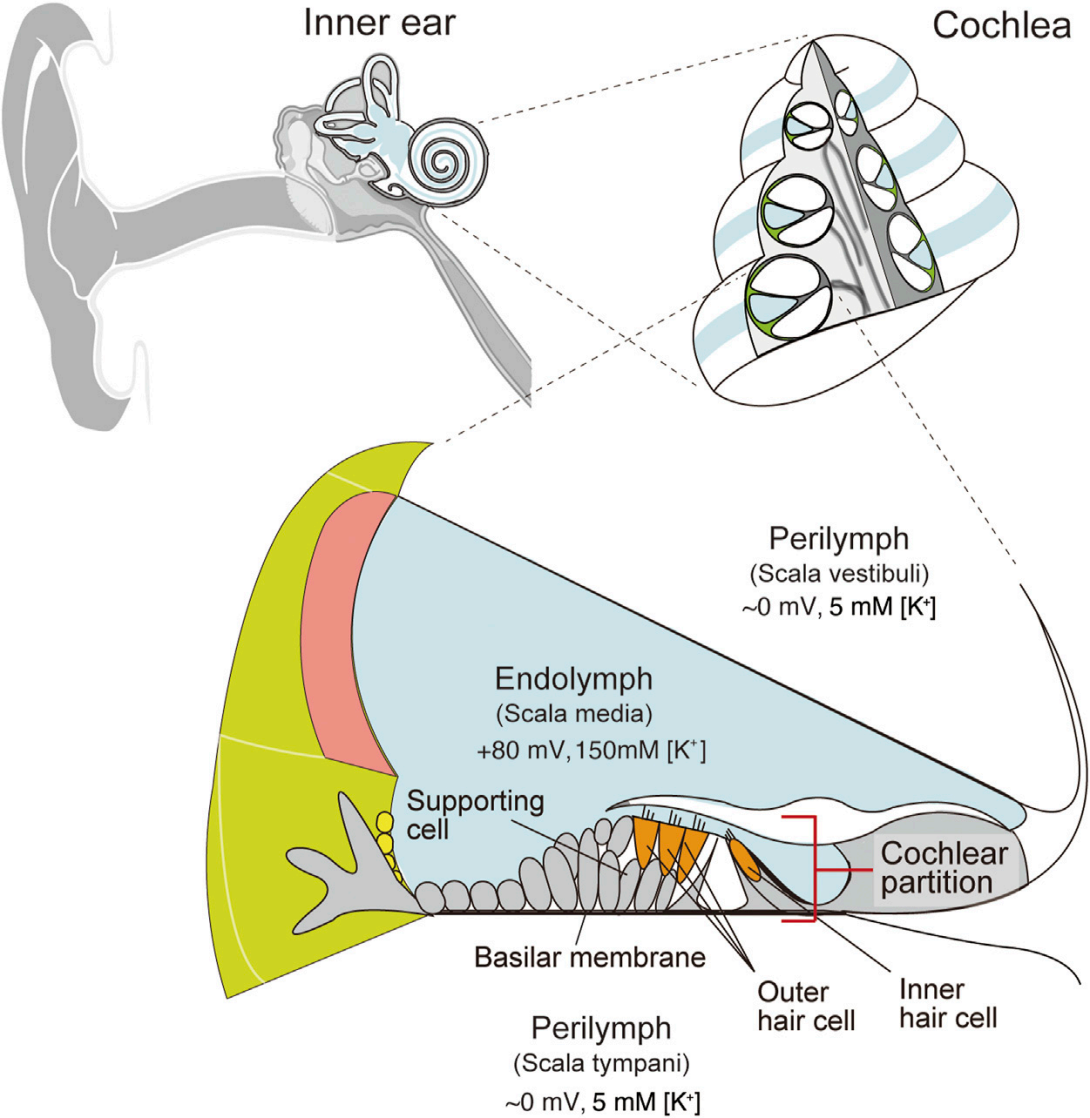
Structure of the cochlea in the inner ear. Schematic images of a human ear and a cross-section of the cochlea are depicted in the upper left and right panels, respectively. The lower panel illustrates the tissue and cellular architecture of the cochlea. Perilymph has ionic composition similar to that of an ordinary extracellular liquid. On the other hand, endolymph maintains 150 mM K^+^ and a highly positive endocochlear potential (EP) relative to perilymph. Cellular composition of the cochlear partition is shown as well. Inner and outer hair cells are surrounded by various types of supporting cells. These cells are mounted on the basilar membrane. Adapted from Nin et al. (2008) with permission. Copyright (2008) National Academy of Sciences.

Audition is an essential sensation for animals. Humans can hear sounds at diverse frequencies ranging from 20 to 20,000 Hz and perceive a millionfold difference in sound pressure level (Hudspeth, 2014). These characteristics stem primarily from the specialized function of the cochlea in the inner ear (Dallos, 1981; Hudspeth, 1989). In mammals, the cochlea has snail-like structure. The interior of this organ is separated by membranous partitions into three chambers: the scala media, scala tympani, and scala vestibuli (Figure 1). The scala media contains a K^+^-rich extracellular solution, endolymph, whereas the scala tympani and scala vestibuli are filled with perilymph, which has ionic composition similar to that of a regular extracellular fluid. Between the scala media and scala tympani lies the “cochlear partition,” which consists of sensory hair cells, supporting cells, and the basilar membrane. Mechanical stimuli of a sound elicit oscillatory waves that travel apically along the cochlear partition. The width of the partition increases, and its thickness and stiffness decrease longitudinally toward the apex from the base (Wever, 1971; Webster and Webster, 1980; Naidu and Mountain, 2007; Ekdale, 2016). This physical property allows a traveling wave to peak in a specific region in accordance with the stimulus frequency; high-pitch sounds induce a motion at the base, whereas low-frequency sounds excite the apex (Békésy and Wever, 1960; Zhang et al., 2007). Vibrations of the cochlear partition next stimulate hair cells, which are classified into inner- and outer hair cells. Both types of cells have their cell bodies immersed in perilymph, whereas their apical surfaces are exposed to endolymph. The mechanical vibrations deflect the hair bundle projecting from the apical surfaces; this event results in the opening of mechanoelectrical transduction channels at the tip of the bundle and permits K^+^ to enter hair cells from endolymph (Oghalai, 2004; Hibino and Kurachi, 2006; Reichenbach and Hudspeth, 2014). Depolarization of inner hair cells induces a neurotransmitter release on the basolateral surface. On the other hand, excitation of outer hair cells changes the length of the cell bodies. This somatic motility can mechanically amplify oscillations of the cochlear partition (Ashmore, 2008; Fettiplace, 2020). The high sensitivity of hearing depends not only on the properties of outer hair cells but also on a highly positive potential of +70 to +90 mV observed in K^+^-enriched endolymph (Békésy, 1952; Dallos, 1996; Hibino et al., 2010; Hudspeth, 2014; Nin et al., 2016). This endocochlear potential (EP) accelerates sound-induced K^+^ entry from endolymph into hair cells: the process that triggers neurotransmission in inner hair cells and somatic motility in outer hair cells. Several studies have shown that the EP is likely to be maintained by ion transport mechanisms in the lateral cochlear wall, which is made up of the stria vascularis and spiral ligament (Wangemann, 2006; Hibino et al., 2010; Nin et al., 2016).

Sensorineural hearing loss (SNHL) is caused by noise exposure, aging, a viral or bacterial infection, or ototoxic drugs (Cunningham and Tucci, 2017). The main organ affected by this disease is the cochlea. In particular, the hearing loss induced by such drugs as loop diuretics, anticancer agents, and antibiotics is a global problem because this adverse effect limits the treatment options and directly worsens the patients’ quality of life during and/or after therapies (Lanvers-Kaminsky et al., 2017). The hearing impairment is reversed by discontinuation of drug administration in many cases but sometimes becomes irreversible due to extensive destruction of cochlear cells, including hair cells. More than 150 drugs have ototoxic effects (Cianfrone et al., 2011; Lanvers-Kaminsky et al., 2017). The measurement of drug pharmacokinetics in the cochlear compartment of live animals would vastly improve our understanding of molecular permeability across the cochlear membranes, and could further aid in improving drug design during lead compound development. It is likely to be difficult to access and accurately analyze the cochlea *in vivo* because this organ is deeply buried in the temporal bone and is composed of fragile membranous and cellular structures. Therefore, relevant pharmacological information is scarce. This problem is also the case for common prescription drugs for the treatment of SNHL; they include corticosteroids, vasodilators, antioxidants, and vitamin preparations. It is noteworthy that although these agents are used empirically for the disease worldwide, their pharmacological targets and clinical significance remain controversial (Rauch, 2008; Agarwal and Pothier, 2009; Schreiber et al., 2010; Wei et al., 2013; Crowson et al., 2017). Accordingly, the pharmacokinetics of ototoxic and therapeutic reagents in the cochlea of the inner ear need to be clarified and have been recently examined by chemical analysis, imaging, and electrochemical procedures. Each of these approaches has its own merits and limitations (Table 1). In the following sections, we describe cochlear pharmacokinetics of different drugs and characteristics of the methodologies used.

**TABLE 1:** Comparison of profiles of different drug detection methods.

Method categories	Method names	Sensitivity	Selectivity	Spatial resolution	Temporal resolution	Live monitoring	Invasiveness	Detection of metabolites	Spatial dimensionality
*In vitro* chemical analysis	HPLC	**++**	**++**	**+**	**+**	±^a^	**+++**	**++**	1 dimension
	LC-MS	**+++**	**+++**	**+**	**+**	±^a^	**+++**	**+++**	1 dimension
	Immunoassay	**+**	**+**	**+**	**+**	±^b^	**+++**	**—**	1 dimension
Imaging	Fluorescence microscopy	**+**	**+** to **++**	**+++**	**+**	±^b^	**++**	**+**	2 dimensions
	Imaging MS	**+++**	**+++**	**++**	**+**	**–**	**++**	**+++**	2 dimensions
	MRI	**+**	**+**	**+**	**++**	**+**	**+**	**—**	3 dimensions
	μCT	**+**	**+**	**++**	**++**	**+**	**+**	**—**	3 dimensions
*In vivo* electrochemistry		**+** to **+++** ^c^	**++**	**++**	**+++**	**+**	**++**	**–** to **++** ^c^	1 dimension

^a^ Possible with microdialysis sampling.

^b^ Possible by means of two-photon laser microscopy.

^c^ These properties vary among different drugs.

## *In Vitro* Chemical Analysis

**FIGURE 2:**
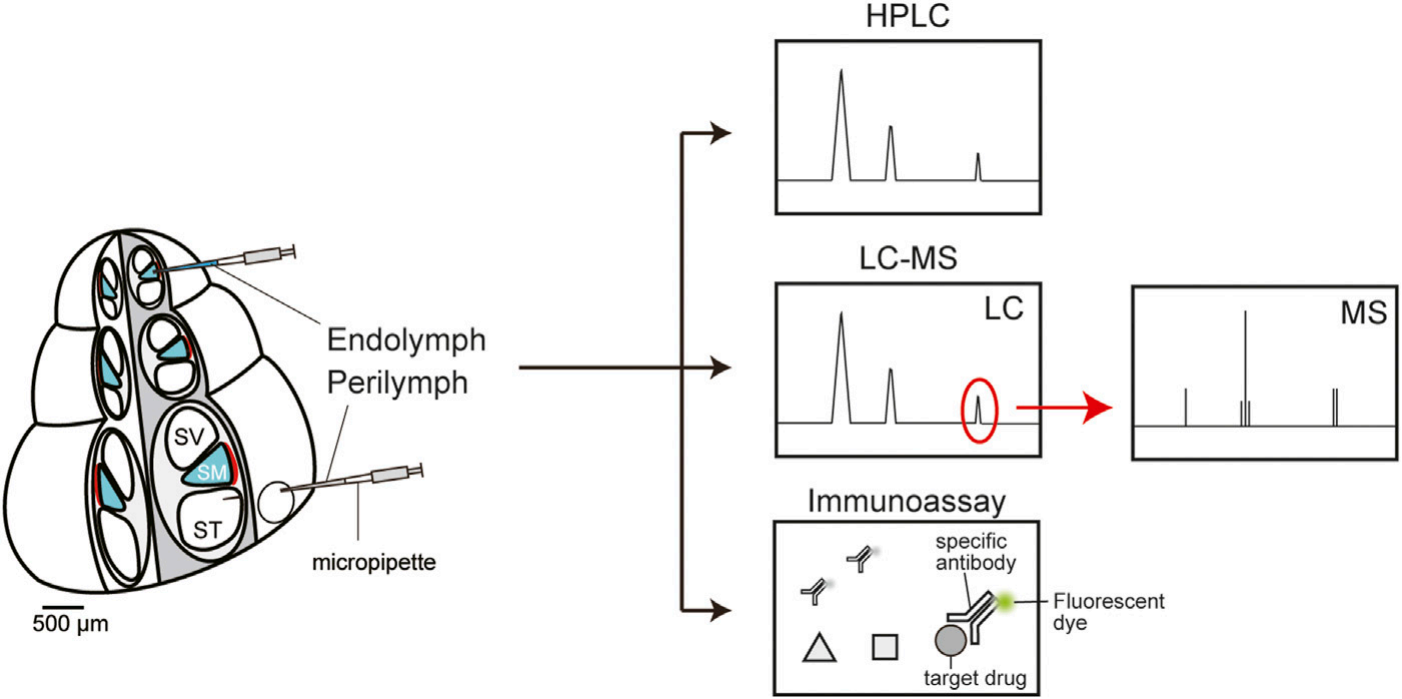
*In vitro* chemical analyses of the perilymph and endolymph. High-performance liquid chromatography (HPLC, top panel) can quickly separate a mixture of molecules from a sample according to their chemical properties such as size and hydrophobicity. Each reagent is sequentially detected as a peak and quantified by calculating the peak area. Liquid chromatography coupled with mass spectrometry (LC-MS, middle panel) is a highly sensitive and selective analytical method for molecular identification and quantification. First, mixtures of multiple compounds are separated by liquid chromatography (LC). Thereafter, the target drug(s) can be extracted and quantified via mass spectrometry (MS). An immunoassay can analyze target molecules using a specific antibody conjugated with a fluorescent dye **(bottom panel)**. This method is characterized by relatively high quantitativity, easy handling, and time- and cost-savings.

To determine drug behavior in the cochlea, in many cases, perilymph and/or endolymph are collected from inner ears of multiple live animals, and the samples are chemically analyzed *in vitro*. High-performance liquid chromatography (HPLC) is a commonly used technique for the quantification of different classes of drugs in the cochlea (Figure 2). The analytes are ototoxic drugs, such as loop diuretics and platinum anticancer drugs, as well as therapeutic agents for deafness, including corticosteroids (Green et al., 1981; Rybak et al., 1984; Laurell et al., 1995; Hahn et al., 2012; Liu et al., 2015). In guinea pigs and chinchillas, drug behavior in cochlear fluids is characterized by a slow time course of the distribution and elimination of the drugs, as compared to the kinetic profiles of plasma and cerebrospinal fluid (Hara et al., 1993; Laurell et al., 1995; Parnes et al., 1999; Hellberg et al., 2009). The relevance of different administration routes to the pharmacokinetics has also been reported; the level of corticosteroids in perilymph after intratympanic administration is significantly higher than that after administration via systemic routes, e.g., intravenous injection or oral gavage (Parnes et al., 1999). Regarding hydrocortisone, maximum concentration (C_max_) and the time to achieve C_max_ (i.e., T_max_) for different administration routes are as follows: 72.42 ± 23.31 mg/L and 1 h for intratympanic injection, 2.03 ± 0.22 mg/L and 1 h for intravenous injection, and 0.86 ± 0.22 at 4 h for oral gavage. In guinea pigs treated with dexamethasone or cisplatin, a small aliquot of cochlear perilymph is aspirated gradually and sequentially via a narrow glass capillary inserted into the apex to determine the longitudinal gradient of the drug concentrations along the cochlea axis. The dexamethasone level in the basal region (136 ± 185 μg/ml) exceeds that in the apical region (∼1 μg/ml) for 2–3 h after intratympanic injection (Plontke et al., 2008). As for cisplatin, its perilymphatic concentration is 4-fold higher in the base than in the apex 10 min after intravenous administration (Hellberg et al., 2013). Notably, this gradient disappears in 30 min. The endolymph in the scala media is more difficult to access and has much smaller volume (∼2 µl) than does the perilymph in the scala tympani and scala vestibuli (∼10 µl) (Shinomori et al., 2001). Nevertheless, this K^+^-enriched fluid has been analyzed in a few studies. Some researchers approached the guinea pig cochlea and quantified corticosteroids in endolymph (Parnes et al., 1999). They demonstrated that a few hours after intratympanic administration, hydrocortisone concentration in endolymph substantially exceeds that in perilymph. Other authors examined the pharmacokinetics of an ototoxic loop diuretic, furosemide, in the endolymph of guinea pigs (Hara et al., 1993). Their assay detected a low concentration and slow time course (C_max_: 1.61 μg/ml, T_max_: 60 min) as compared to the profile in perilymph (C_max_: 4.9 μg/ml, T_max_: 15 min). Pretreatment with probenecid, an inhibitor of the anion transporters mediating active transport of furosemide, drastically delayed the delivery of the drug into perilymph, whereas this arrangement had a negligible effect on the pharmacokinetics in endolymph and serum. This result suggests that furosemide is passively imported into endolymph through a blood vessel with relatively low permeability.

Liquid chromatography coupled with mass spectrometry (LC-MS) has the advantage of highly sensitive detection of drugs as well as accurate separation of compounds with similar structure, e.g., metabolites (Figure 2). With this method, perilymph in guinea pigs was analyzed to clarify the pharmacokinetics of dexamethasone administered via three different routes: intraperitoneal, intratympanic, and postaural injection (Wang et al., 2018). The intratympanic administration yielded a higher maximum drug concentration within a shorter period (C_max_: 25,343.38 ± 2641.17 ng/ml, T_max_: 1 h) than did the other two routes (C_max_: 532.56 ± 145.66 ng/ml, T_max_: 4 h for postaural injection; C_max_: 100.87 ± 48.05 ng/ml, T_max_: 2 h for intraperitoneal injection). Some investigators addressed how a difference in the dosing route can affect drug pharmacokinetics in the rat cochlea, as follows (Grondin et al., 2013): They administered antioxidant d-methionine to animals intratracheally, intranasally, orally, or intravenously; sampled a cochlear fluid containing both perilymph and endolymph; and examined the compounds’ behavior by LC-MS. The pulmonary delivery induced the highest concentrations of methionine (C_max_: 110.3 ± 27.6 µM) and its metabolite acetyl-l-carnitine (C_max_: 12.9 ± 12.5 µM), whereas the intranasal route was the least effective: C_max_ of 12.0 ± 7.4 µM for methionine and 2.9 ± 0.3 µM for the metabolite.

Immunoassays for drug quantification are currently employed not only for basic research but also for clinical tests (Figure 2). In general, the advantages are relatively high quantitativity, easy handling, and time- and cost savings (Stead, 2000; Taylor et al., 2015). Nevertheless, sensitivity and selectivity of these methods are lower than those of HPLC and MS. Moreover, their applications are limited to the drugs against which antibodies have been developed. Specific antibodies are available against several antibiotics including vancomycin, amikacin, and gentamicin. As for the cochlea of guinea pigs and chinchillas, a fluorescence polarization immunoassay clarified the kinetics and longitudinal gradient of ototoxic gentamicin in perilymph (Hoffer et al., 2001). In chinchillas, a continuous release of the drug toward the round window membrane through a microcatheter controlled by an osmotic pump resulted in lower C_max_ and longer retention time than did intratympanic administration (Hoffer et al., 2001). A peak concentration of 913 mg/ml was registered 24 h after transient injection of 3.75 mg gentamicin sulfate, and then the drug was quickly eliminated, within 48 h. On the other hand, sustained delivery of a 10 mg/ml drug solution at a rate of 1 μl/h led to nearly identical concentrations at 24, 48, and 72 h, with a peak of 322 mg/ml (72 h). In a guinea pig cochlea into which gentamicin was administered from the round window membrane, a large concentration gradient along the longitudinal axis was found; the concentration was >4000-fold higher at the base than at the apex (Plontke et al., 2007). Conversely, after systemic application of intravenous and subcutaneous dosing, the perilymphatic drug level was highest at the apex and gradually decreased as the analyzed point was moved toward the basal region (Hahn et al., 2013).

All the analytical methods described above require a few invasive procedures for animal preparation. First, because the cochlea is deeply buried in the temporal bone, a surgical procedure to approach this organ is mandatory. Further, it is necessary to make a fenestra on the cochlear bony wall to sample cochlear fluids. Additionally, the sampling requires insertion and indwelling of pipettes or tubes. Another problem is that excess collection of perilymph or endolymph is likely to impair physiological function of the cochlea and to contaminate such fluids with neighboring blood and cerebrospinal fluid. Owing to this issue as well as the extremely small total volume of perilymph (∼10 μl) and endolymph (∼2 μl; guinea pig), only a limited amount of cochlear fluids can be obtained. In this context, highly sophisticated and expert skills are necessary to implement stable and reproducible experiments. Most investigators validate the purity of a sample and/or intactness of cochlear function during fluid aspiration by measuring perilymphatic and/or endolymphatic physiological parameters such as ion concentrations and a potential (Green et al., 1981; Rybak et al., 1984; Hara et al., 1993; Parnes et al., 1999). On the basis of such observations, it seems probable that cochleostomy and contamination with cerebrospinal fluid during sample collection considerably affect drug kinetics (Salt and Plontke, 2005). An additional concern is relatively low spatial resolution. The conventional methods require certain amounts of samples and therefore necessitate the collection of perilymph or endolymph compartments that may originate from a wide range of cochlear chambers. As described above, a few research groups have examined the longitudinal gradient of drug distribution in the guinea pig cochlea (Plontke et al., 2008; Hellberg et al., 2013; Salt et al., 2016). In these cases, perilymph was sequentially sampled from a hole made in the cochlear apex such that the fluid was sorted into 10 or fewer segments along the longitudinal axis. Spatial resolution of this procedure may be worse than 1 mm because the total longitudinal length is approximately 18 mm (Shinomori et al., 2001). It is also crucial that the number of samplings of cochlear fluids be practically restricted to once or twice in an individual animal. Therefore, a large number of animals should be examined to determine the kinetics of a drug over time. In this regard, significant differences in the data are observed among different animals, and this interanimal variation can interfere with accurate determination of pharmacokinetics (Hahn et al., 2006). This disadvantage further worsens time resolution of the kinetic data. The T_max_ values of the majority of drugs are in the scale of dozens of minutes to several hours when administrated to living animals systemically (Rowland and Tozer, 2005; Brunton et al., 2018). Therefore, ideally, the drug concentration should be measured every 2–10 min for a few hours or even for a few days to accurately understand the kinetics. Nonetheless, to achieve this resolution with the conventional methods, a large number of experimental animals is required. Accordingly, in most studies, the data are collected at an interval of dozens of minutes or hours. These problems may be at least in part overcome by a microdialysis technique that can continuously obtain biological samples from an individual animal (Chaurasia et al., 2007). Nevertheless, the method remains invasive because it should be accompanied by insertion of a sampling probe with a diameter of >200 µm into the cochlea; this technique may damage tissue structure and affect fluid flow (Khan and Michael, 2003). In addition, considerable prolonged sample collection is likely necessary to ensure the quantitativity of drug concentration.

In summary, HPLC, LC-MS, and an immunoassay with sampled perilymph and endolymph are the methods frequently used for quantifying drug concentrations in these cochlear fluids over time. Although such conventional methods have provided key insights into the pharmacokinetics of a few ototoxic and therapeutic reagents, the shortcomings to be addressed are the invasiveness associated with the sampling, low spatiotemporal resolution, and necessity of a large number of animals for the determination of longitudinal drug behavior.

## *In Vivo* Imaging Techniques

**FIGURE 3:**
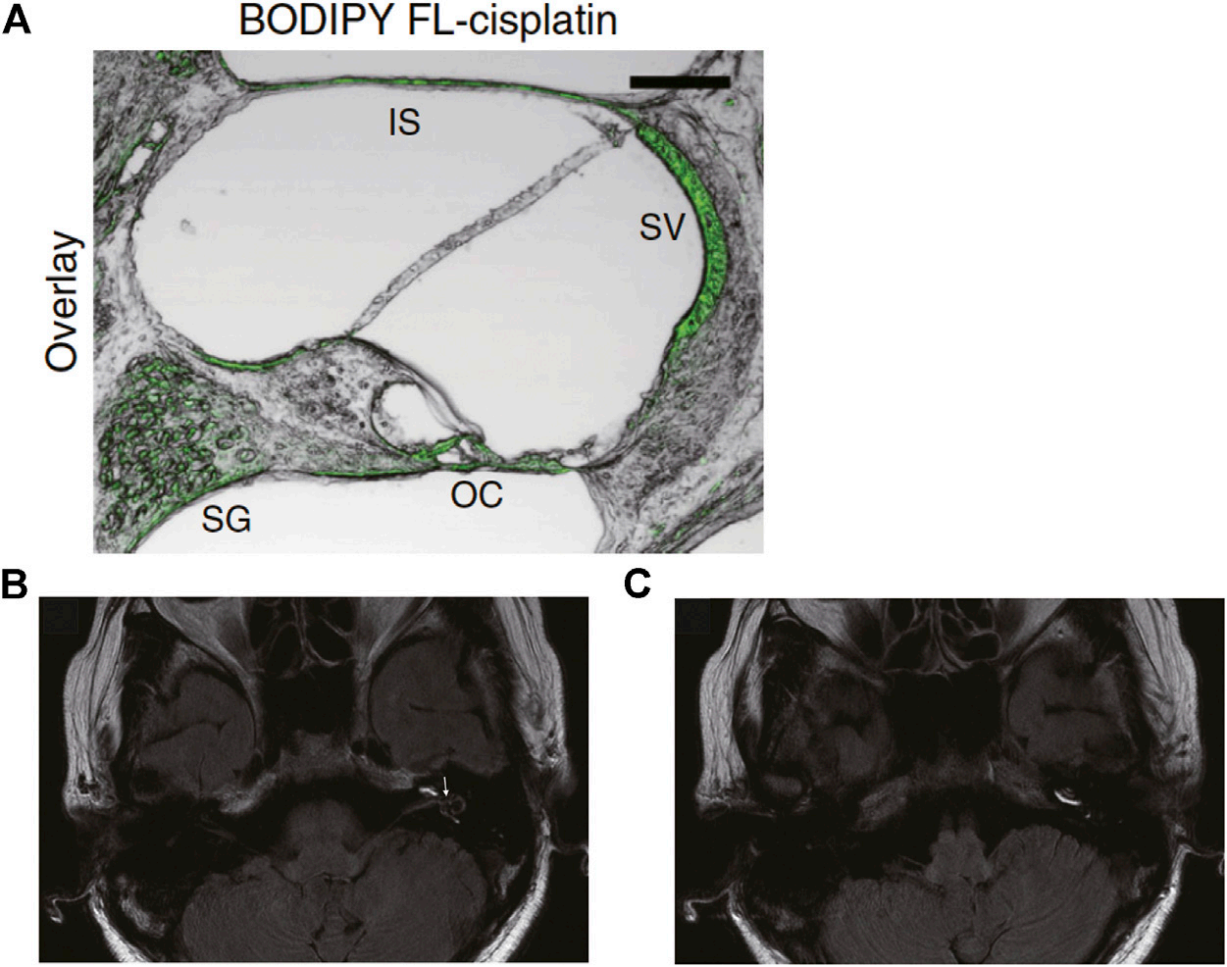
*In vivo* imaging analyses of the cochlea. **(A)** Confocal image of a slice from cochlear cryosections of mice systemically administered cisplatin conjugated with a green fluorescent dye, BODIPY FL. The green signal indicates cisplatin accumulation. SG: spiral ganglion, OC: organ of Corti, SV: stria vascularis, IS: interscalar septum. Adapted from Breglio et al. (2017) with permission from Springer Nature. Copyright (2017) Nature Communications. **(B,C)** Three-dimensional magnetic resonance images taken 1 day after intratympanic injection of gadolinium in a patient with Meniere’s disease. **(B)** Gadolinium signals are observed in the perilymphatic area of the basal and second turns of the cochlea, as well as in the vestibule and semicircular canals. Large black areas in the vestibule represent vestibular endolymphatic hydrops (arrow). **(C)** The same image scanned at a different cross-section. The scala tympani of the three turns of the cochlea are clearly visualized by gadolinium. Adapted from Nakashima et al. (2007) with permission from John Wiley and Sons. Copyright (2007) The Laryngoscope.

Molecular imaging is widely utilized to analyze biological phenomena and is applied in pharmacological research. Recent progress in optics and data-processing techniques offers a variety of options. Fluorescence imaging using confocal microscopy is a common method. This approach is characterized by the high spatial resolution of several hundred nanometers and can determine the cellular distribution or subcellular localization of drugs in biological samples. In the cochlea, the distribution of dexamethasone was visualized as follows: mice were treated with dexamethasone via hypodermic or intratympanic routes, and the distribution and pharmacokinetics in cochlear slice sections were examined (Grewal et al., 2013). The drug was immunohistochemically detected with a specific antibody and a secondary antibody conjugated with a fluorescent molecule. Fluorescence was well pronounced in inner hair cells, and this cellular signal gradually decreased from the base toward the apex along the cochlear axis. In addition, dexamethasone injected *via* the transtympanic route was retained in the hair cells for the long period of 12 h, whereas the systemically administered drug was rapidly eliminated from the cells, with weak staining at 6 h and almost no signal after 12 h. In rats, an intracochlear distribution of dexamethasone was determined (Lee et al., 2018); in that study, the compound was directly labeled with fluorescein isothiocyanate. Obvious signals were noted in the spiral ganglion, organ of Corti, and lateral cochlear wall 6 h and 3 days after intraperitoneal or intratympanic injection. Furthermore, in these tissue components, the drug was likely to be retained for 7 days after intratympanic administration, whereas at that time point, only a small proportion remained there in the case of systemic application. Distribution and pharmacokinetics of gentamicin and cisplatin are examined by fluorescence imaging as well. One research group assayed gentamicin in the inner ear of chinchillas by an immunolabeling approach and analyzed the difference in the effect on the drug behavior between intratympanic injection and a sustained release from an osmotic pump (Roehm et al., 2007). Cochlear samples were evaluated at 4 and 8 h. With either administration route, the fluorescent signal was concentrated on the spiral ganglion, lateral wall, and organ of Corti. A minimal concentration gradient was observed along the longitudinal axis of the cochlea. In another work, fluorescent-dye–conjugated cisplatin was used to determine the cellular distribution in the mouse cochlea (Figure 3A) (Breglio et al., 2017). The procedure for preparation of the cisplatin ototoxicity model was as follows: The mice underwent three cycles of cisplatin treatment; each cycle consisted of intraperitoneal drug administration at 3.5 mg/kg per day for four consecutive days, followed by a recovery period of 10 days. After the completion of the regimen, a fluorescent signal was well pronounced in the stria vascularis and moderately strong in the spiral ganglion, organ of Corti, and interscalar septum, a bony plate that separates adjoining cochlear turns.

The imaging approaches mentioned above provide crucial insights into drug distribution, which is valuable for understanding the mechanisms underlying desired and adverse therapeutic effects. Nevertheless, some disadvantages should be considered. In general, this method is applied to slice sections of cochlear tissues dissected from euthanized animals. Therefore, only single-timepoint data can be obtained from an individual animal. Furthermore, in many cases, the cochlea is perfused with phosphate buffers and/or fixation agents before sectioning. This pretreatment washes out the drugs from the scala tympani, scala vestibuli, and/or scala media to some extent. These shortcomings can be addressed by two-photon excitation microscopy, which employs highly tissue-penetrating long-wavelength types of light and thus enables live and deep-tissue imaging. Nevertheless, readers should keep in mind that exposure to high-power laser pulses for a long period can have deleterious effects, including photobleaching and heating (Huisken and Stainier, 2009). The other problem for fluorescence imaging is relatively low quantitativity as compared to *in vitro* techniques such as HPLC and MS. In addition, it cannot be ruled out that labeling a drug with fluorescent molecules can affect its properties related to pharmacokinetics and tissue distribution.

Imaging mass spectrometry is an advanced technique that extends mass spectrometry to two-dimensional mapping of substances in biological samples. This approach can quantitatively determine a spatial distribution of a broad array of molecules, such as drugs, proteins, and their metabolites, at high resolution (McDonnell and Heeren, 2007). A practical example is a cisplatin distribution in a cochlear slice section in mice (Breglio et al., 2017). Although imaging mass spectrometry requires no labeling procedure for the compounds, the samples need to be prepared from the cochleae extracted from euthanized animals. Therefore, pharmacokinetics cannot be assessed over time in a live animal.

Noninvasive three-dimensional imaging technologies such as magnetic resonance imaging (MRI) and micro–computed tomography (μCT) are available for physiological and pharmacological studies. MRI can acquire information on tissue structures and functions by measuring the signals generated by magnetic resonance of certain atomic nuclei. A contrast agent, gadolinium hydrate, was injected intratympanically into patients with an inner ear disorder, Meniere’s disease, and the distribution was monitored over time to track the sizes of the cochlear chambers, scala tympani, and scala vestibuli (Nakashima et al., 2007). With this technique, endolymphatic hydrops was detected clearly (Figure 3B). The behavior of the injected contrast agent was analyzed too; gadolinium first went into the basal turn of the scala tympani and then spread to almost all parts of perilymphatic space 1 day after the injection (Figure 3C). In rats, the perilymphatic space was visualized through 9.4 T µMRI imaging. The cochlea was scanned every 30 min from the timepoint of 1–4 h after intratympanic injection of either gadoterate meglumine or gadodiamide (Park et al., 2016). The perilymphatic space, but not endolymphatic space, was clearly analyzed in live animals. Note that in >50% of the tested cochleae, a significant inflammatory response was found in the scala tympani with either contrast agent. CT can yield tomographic images of an object via multiangle X-ray scanning. Combining the multiple images results in the reconstruction of three-dimensional structure of the object. μCT is a specialized technique that is characterized by high spatial resolution and is applicable to tiny biological samples. A few researchers have taken the challenge of μCT imaging of cochlear fluids in live animals. For instance, in mice, artificial perilymph containing an iodine-based contrast agent, ioversol, was continuously perfused into the cochleae from the scala tympani at the basal turn, while μCT imaging was performed on the organ (Haghpanahi et al., 2013). A series of scans took 13.2 min, which visualized the anatomy of the whole cochlea (voxel size: 15 × 15 × 15 μm). From signal intensities in each pixel of the image, the distribution and kinetics of the agent were determined over ∼90 min within scala tympani and scala vestibuli. As expected, the compound’s concentration was the highest at the base of the cochlea. In addition, the longitudinal gradient of the concentration stabilized 55 min after the infusion. Another research group performed μCT imaging to examine the kinetic profile of iopamidole in the mouse cochlea (Moudgalya et al., 2019). This contrast agent was infused from the scala tympani at the cochlear basal end. In each experiment, the imaging was started after ∼30 min of iopamidole infusion. Five contrast scanning images were continuously acquired during 20 min. The temporal distribution of the agent along the longitudinal axis was quantified in the scala tympani, scala media, and scala vestibuli by using reconstructed three-dimensional images. By means of this spatiotemporal profile, diffusion and transport parameters of the agent were estimated to be: the diffusion coefficient at zero concentration (D_0_) = 6.226 × 10^–4^ mm^2^/s and transport coefficients between the scala tympani and scala media (K_ST-SM_) = 1.01 × 10^–3^ mm/s, between the scala media and scala vestibuli (K_SM-SV_) = 0.38 × 10^–3^ mm/s, and between scala media and a clearance compartment (K_SM-clearance_) = 0.035 × 10^–3^ mm/s. This information was used to develop a cochlear pharmacokinetic model.

So far, the application of the three-dimensional live imaging mentioned above has been restricted to the quantitation of contrast agents per se. This approach may be applied to different ototoxic or therapeutic drugs from the standpoint of minimally invasive pharmacokinetic studies in live animals. Nonetheless, there are a few limitations to this method. Firstly, temporal resolution is still insufficient because multiple scanning for three-dimensional reconstruction of the object takes 10–30 min. Furthermore, these techniques require labeling of the drugs; this modification may alter the kinetics, distribution, and pharmacological effects in some cases. Besides, it is difficult for this methodology to separate the signals of parent drugs from the signals of their metabolites.

## *In Vivo* Electrochemistry

**FIGURE 4:**
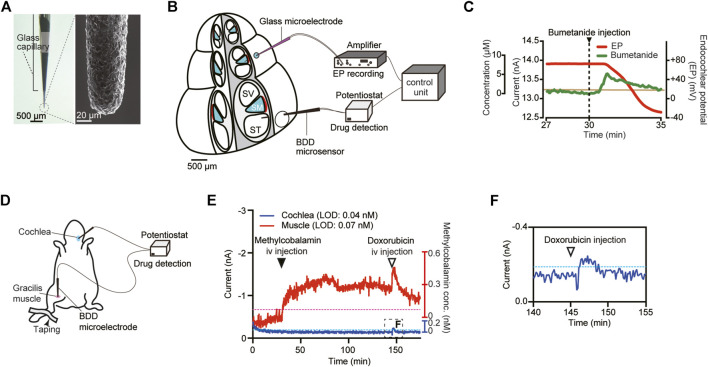
*In vivo* electrochemical drug monitoring with a boron-doped diamond (BDD) electrode. **(A)** A needle-type BDD microelectrode. As shown in the left panel, the BDD needle was encased with a glass capillary tube. The needle tip, which has a diameter of ∼40 μm, was pulled out of the capillary. The right panel displays a magnified image of the tip obtained by means of a scanning electron microscope. **(B)** The experimental setup for *in vivo* detection of bumetanide in a cochlea. A BDD microelectrode was inserted into the perilymph of the scala tympani (ST), whereas a glass microelectrode for recording of the endocochlear potential (EP) was placed in the endolymph of the scala media (SM). SV: scala vestibuli. **(C)** *In vivo* recording of bumetanide levels in the cochlea. Bumetanide was injected into the external jugular vein 30 min (arrowhead) after the onset of the measurement. The green line denotes the current detected by the BDD microelectrode, whereas the EP measured with the glass microelectrode is displayed by the red line. The concentration scale on the vertical axis was calculated on the basis of *in vitro* calibration of the BDD electrode (for details, see Ogata et al., 2017). The brown line means the *in vivo* limit of detection for the microelectrode. Adapted from Ogata et al. (2017) with permission from Springer Nature. Copyright (2017) Nature Biomedical Engineering. **(D)** Schematic illustration of the *in vivo* experimental setup for methylcobalamin detection in a guinea pig. The system contains two different BDD microsensors: one was inserted into cochlear perilymph, and the other was placed in the extracellular space of the gracilis muscle of the right hind leg. **(E,F)** *In vivo* recording of methylcobalamin concentrations. The currents in perilymph (blue) and in the muscle (red) were simultaneously measured with two BDD microelectrodes. The concentration scales on the vertical axis are derived from *in vitro* calibration for each electrode (for details, see Hanawa et al., 2020). Methylcobalamin (10 mg/kg) was injected into the left external jugular vein at 30 min (filled arrowhead) after the onset of the recording. Then, doxorubicin (5 mg/kg) was injected via the same route (open arrowhead). The trace in the boxed region in **(E)** is enlarged in panel **(F)**. The limit of detection (LOD) for each microsensor is indicated as a blue (muscle) or red (cochlea) dotted line. Adapted from Hanawa et al. (2020) with permission. Copyright (2020) American Chemical Society.

Electrochemistry with the help of small and sharp electrodes is a powerful analytical method for detecting substances *in vivo*. In this procedure, the electrode is directly exposed to a solution containing chemical compound(s); application of suitable potentials to this sensor causes a redox reaction of the compound. The reaction is accompanied by a transfer of electron(s), whose number depends on the concentration of the reactant. Therefore, the detected current density is commensurate with the concentration of the compound (Bagotsky, 2005). The conventional materials utilized for constructing the electrodes are gold, platinum, and some carbon types including glassy carbon. A major application of this technique is the monitoring of neurotransmitters in the brain (Venton and Wightman, 2003; Jackowska and Krysinski, 2013); a limited number of investigators have examined the cochlea. An example is real-time measurement of ascorbate concentration in the perilymph of guinea pigs by means of carbon fiber microelectrodes modified with multiwalled carbon nanotubes (Liu et al., 2012). This experiment showed that when ototoxic salicylate was injected into perilymph through a microinfusion system, ascorbate concentration sharply decreased to 28 ± 10% (45.0 ± 5.1 μM) of its baseline level in 2.5 min. The other approach is a microsensing system that we recently developed (Ogata et al., 2017). The sensor here is a needle-type electrode composed of boron-doped diamond (BDD), which is an advanced material for electrochemistry (Figure 4A), and this sensor was employed for *in vivo* real-time detection of a drug. BDD has several prominent advantages (Einaga et al., 2001; Fujishima et al., 2005). First, this material offers high physicochemical stability and response speed and thus enables prolonged recording with high temporal resolution. Additionally, a BDD electrode can detect a broad array of compounds due to the wider potential window of water stability as compared with that of classic materials such as carbon, platinum, and gold. Moreover, the background noise induced by the reaction on the BDD electrode is low. These characteristics depend primarily on the properties of the electrode’s surface, which is composed predominantly of sp^3^ carbon; this material reduces the adsorption of reactants to the electrode (Fujishima et al., 2005). In our microsensing system, the needle-type BDD microelectrode was combined with a glass microelectrode, which can monitor cellular electrical activity in a small area (Ogata et al., 2017). Drug behavior can be determined every 5 s, thereby providing high temporal resolution for pharmacokinetic studies. As a test analyte, we selected the ototoxic reagent bumetanide; this loop diuretic can reduce the EP in endolymph and thus induce hearing loss (Figure 4B). The BDD microelectrode, when placed in perilymph, detected a clear-cut and rapid response with T_max_ of ∼1.5 min and C_max_ of 5.3 µM in an experiment shown in Figure 4C. On the other hand, the EP measured by the glass microelectrode inserted into endolymph began to decrease when bumetanide concentration was maximized. Finally, the EP reached a negative value of –30 mV, which mirrors severe hearing impairment, although the signal detected by the BDD electrode returned to baseline. It is noteworthy that the drug kinetics were clearly different from the EP kinetics, which correlate with the hearing threshold. These results indicate that our microsensing system can simultaneously monitor pharmacokinetics and pharmacodynamics in a live cochlea. Additionally, we constructed a different system, composed of two BDD microelectrodes, and examined methylcobalamin concentration in separate targets (Hanawa et al., 2020). This cobalamin derivative is often administered to patients with SNHL (Wang et al., 2013). In an anesthetized guinea pig, one microsensor was inserted into cochlear perilymph in scala tympani, whereas the other was placed in the extracellular space of a leg muscle (Figure 4D). Then, a solution containing 10 mg/kg methylcobalamin was injected into the left external jugular vein. Figure 4E shows a representative recording. Over a period of 120 min, little change in the current was observed in perilymph. By contrast, a clear response was detectable in the leg muscle; current amplitude markedly increased immediately after the injection and reached a peak of 0.4 μM in 45 min. As a control experiment, 5 mg/kg doxorubicin, which can react on a BDD microelectrode (Ogata et al., 2017), was additionally injected at a time point of 150 min (Figures 4E,F). As expected, a rapid response was detectable in both perilymph and the leg muscle. Although the amount of the systemically injected methylcobalamin (10 mg/kg) greatly exceeded the clinical dose (10 μg/kg) (Yamane et al., 1995), a current exceeding the detection limit was not detectable in cochlear perilymph. These findings suggest that methylcobalamin possibly reaches compartments other than perilymph and exerts a pharmacological effect.

As described above, the electrochemical approach can help to monitor concentrations of substances *in vivo* with high sensitivity. This method can target a small region—even inside cochlear chambers—with a sharp sensor so that it can minimize contamination with off-target body fluids and cells. Nevertheless, the surgical procedures for exposure of the cochlea and for making a hole for electrode insertion on the cochlear bony wall are invasive for animals to some extent and may modestly but significantly affect hearing. Another limitation is that certain types of chemical compounds are electrochemically inactive. Moreover, with a needle-type microelectrode, two-dimensional mapping of drug distribution is impossible.

## Discussion

The determination of pharmacokinetics of ototoxic and therapeutic drugs in the cochlea should provide key insights into the mechanisms underlying drug-induced hearing loss and may facilitate the development of effective therapies for SNHL. Nonetheless, this task is still challenging due to technical hurdles. As the cochlea has a complicated structure and a “blood–labyrinth barrier” that interferes with drug permeation, it is difficult to predict the pharmacokinetics in a target small area inside the organ. The majority of pharmacokinetic studies in the cochlea have analyzed the perilymph. In this regard, these observations provide valuable insights into the permeability of a few drug molecules across the vessels and cellular membranes to the perilymph. Nevertheless, little is known about the transport of compounds into the endolymph. Furthermore, it remains largely unclear whether and how the behavior of a drug in these cochlear fluids is correlated with its distribution within the cellular and tissue components of the cochlear partition and lateral wall. The analytical procedures described in this review have various advantages and disadvantages. In addition to technical improvements, the combination of multiple methods will help to clarify unexpected drug behavior in the “labyrinth.”
